# Association of the Recessive Allele *vrn-D1* With Winter Frost Tolerance in Bread Wheat

**DOI:** 10.3389/fpls.2022.879768

**Published:** 2022-06-06

**Authors:** Hongjun Zhang, Xinhui Xue, Jie Guo, Yiwen Huang, Xuran Dai, Teng Li, Jinghuang Hu, Yunfeng Qu, Liqiang Yu, Chunyan Mai, Hongwei Liu, Li Yang, Yang Zhou, Hongjie Li

**Affiliations:** ^1^Institute of Crop Sciences, Chinese Academy of Agricultural Sciences, National Engineering Research Center of Crop Molecular Breeding, Beijing, China; ^2^College of Life Sciences, Shanxi University, Taiyuan, China; ^3^College of Agriculture, Shanxi Agricultural University, Jinzhong, China; ^4^College of Agronomy and Biotechnology, Hebei Normal University of Science and Technology, Qinhuangdao, China; ^5^Zhaoxian Experiment Station, Shijiazhuang Academy of Agricultural and Forestry Sciences, Shijiazhuang, China; ^6^Xinxiang Innovation Center for Breeding Technology of Dwarf-Male-Sterile Wheat, Xinxiang, China

**Keywords:** winter frost damage, marker-assisted selection, cold-responsive genes, grain yield, genomic composition

## Abstract

Winter frost has been considered the primary limiting factor in wheat production. Shimai 12 is an elite wheat cultivar grown in central and southern Hebei province of China, but sensitive to winter frost. In this study, the winter frost tolerant cultivar Lunxuan 103 was bred by introducing the recessive allele *vrn-D1* from winter wheat Shijiazhuang 8 (frost tolerance) into Shimai 12 using marker-assisted selection (MAS). Different from Shimai 12, Lunxuan 103 exhibited a winter growth habit with strong winter frost tolerance. In the Shimai 12 × Shijiazhuang 8 population, the winter progenies (*vrn-D1vrn-D1*) had significantly lower winter-killed seedling/tiller rates than spring progenies (*Vrn-D1aVrn-D1a*), and the consistent result was observed in an association population. Winter frost damage caused a significant decrease in grain yield and spike number/m^2^ in Shimai 12, but not in Lunxuan 103 and Shijiazhuang 8. The time-course expression analysis showed that the transcript accumulation levels of the cold-responsive genes were higher in Lunxuan 103 and Shijiazhuang 8 than in Shimai 12. Lunxuan 103 possessed the same alleles as its parents in the loci for plant height, vernalization, and photoperiod, except for the vernalization gene *Vrn-D1.* An analysis of genomic composition showed that the two parents contributed similar proportions of genetic compositions to Lunxuan 103. This study provides an example of the improvement of winter frost tolerance by introducing the recessive vernalization gene in bread wheat.

## Introduction

Low-temperature stress is the primary limiting factor that determines the growth, development, and geographical distribution of wheat (*Triticum aestivum* L.) (Laudencia-Chingcuanco et al., 2011; Vítámvás et al., 2019). Frost damage occurs frequently in northern and Eastern Europe, North America, and China, and greatly threatens wheat production (Babben et al., 2018; Muhammad et al., 2021). Tolerance to low temperatures is essential for the fall-planted temperate cereals to survive the freezing temperatures during the winter season (Dhillon et al., 2010). Winter wheat is usually planted in autumn and must have substantial tolerance to winter frost during winter and resumes normal growth in the next spring (Galiba et al., 2009; Case et al., 2014). Different from winter frost, spring radiation frost usually takes place at the late vegetative and reproductive stages and results in anther sterility, floret, spike abortion, and damage to developing grains (Xiao et al., 2018; Muhammad et al., 2021).

Three groups of genes conferring vernalization (*Vrn*), photoperiod (*Ppd*), and *CBF* (C-repeat binding factors) transcription factors determine the frost tolerance of wheat (Galiba et al., 2009; Janmohammadi and Mahfoozi, 2013; Babben et al., 2018). Vernalization is a physiological process for winter and biennial species, which requires exposure to a period of low temperature to generate flowering competence (Xu and Chong, 2018; Xu S. et al., 2021). The response to vernalization in wheat is mainly controlled by five genes, *Vrn-A1*, *Vrn-B1*, and *Vrn-D1* on chromosomes 5A, 5B, and 5D, *Vrn-D4* on chromosome 5D, and *Vrn-B3* on chromosome 7B (Fu et al., 2005; Yan et al., 2006; Kippes et al., 2015). The alleles at locus *Vrn-D1* control three types of growth habits, in which the dominant alleles *Vrn-D1a* and *Vrn-D1b* confer the spring and facultative growth habits, respectively, and the recessive allele *vrn-D1* is related to the winter growth habit (Fu et al., 2005; Zhang et al., 2012). Two dominant alleles share the same deletion in the first intron but have a single nucleotide polymorphism (SNP) mutation at the −161 bp position in the promoter region (Zhang et al., 2012).

Low-temperature acclimation is a process that makes plants acquire winter frost tolerance after exposure to nonfreezing temperatures (Shi et al., 2018). The requirement for nonfreezing low temperature is common to both low-temperature acclimation and vernalization, indicating the potential connection between the two processes (Dhillon et al., 2010; Xu and Chong, 2018). Winter wheat shows a clear decline in frost tolerance when the requirement of vernalization is met (Linmin and Fowler, 2006). Several major QTL *Fr-A1*, *Fr-B1*, and *Fr-D1* for wheat frost tolerance are located on the same homeologous group 5 chromosomes as the vernalization genes *Vrn-A1*, *Vrn-B1*, and *Vrn-D1* (Sutka and Snape, 1989; Snape et al., 2001; Sutka, 2001; Tóth et al., 2003; Babben et al., 2018). Previous studies have found that wheat cultivars with the dominant *Vrn-A1a* or *Vrn-A1b* alleles were more sensitive to low temperature than those carrying the dominant *Vrn-B1* or *Vrn-D1* alleles, but they were more sensitive than the cultivars carrying the recessive alleles (Koemel et al., 2004; Reddy et al., 2006). The recessive allele *vrn-A1* can increase freezing tolerance between 2.1- and 2.4-folds in winter and spring wheat compared to the dominant allele *Vrn-A1*, respectively (Linmin and Fowler, 2006). These results suggest that the vernalization genes play an important role in the regulation of winter frost tolerance, and the recessive *vrn-1* allele is strongly associated with frost tolerance.

The Yellow and Huai River Valley Winter Wheat Zone (YHWWZ) is the largest wheat-growing region in China, where frost damage has been one of the primary constraints to wheat production (Zhao et al., 2020). Hebei province is located across two wheat ecological zones, that is, the Northern Winter Wheat Zone (NWWZ) in the northern part and the YHWWZ in the southern part of the province (Li H.J. et al., 2019). Frost damage caused by low temperature during long-term winter mainly occurs in northern Hebei, whereas sudden temperature decline in early winter mainly occurs in the central-southern province (Dai et al., 2014). In the central and southern Hebei, some cultivars grow fast in warm winter due to less vernalization requirement. A sudden drop in ambient temperature at the seedling stage causes extensive winter frost injury and losses in grain yield. The most serious winter frost resulted in winterkill damage in 30% of the wheat acreage in that region in 2009 (Wang et al., 2013). It is necessary to improve winter frost tolerance in order to warrant safe production in this area.

Shimai 12, a wheat cultivar with high-yielding potential and early maturity, was commercially released as a facultative wheat cultivar in 2004 (Wu, 2005). However, it was withdrawn from production due to its sensitivity to winter frost causing serious economic losses in 2005 and 2009 (Wang et al., 2013). Shimai 12 shows spring growth habit and carries the dominant *Vrn-D1a* allele at the *Vrn-D1* locus, indicating that it is a typical spring wheat cultivar (Zhang et al., 2010, 2012). Hence, the poor winter frost tolerance of Shimai 12 is probably attributed to its spring growth habit.

The present study was carried out to determine the effectiveness of *Vrn-D1* in improving winter freezing tolerance by introducing the recessive *vrn-D1* allele at the *Vrn-D1* locus into Shimai 12 resulting in the development of Lunxuan 103.

## Materials and Methods

### Plant Materials

In 2003, Shijiazhuang 8 (Jimai 38/Shi 91-5065, *vrn-D1vrn-D1*), a high-yielding winter wheat cultivar with better winter frost tolerance and high kernel weight, was crossed to a spring wheat cultivar Shimai 12 (Shi 91-5096/Jimai 23, *Vrn-D1aVrn-D1a*). The F_1_ plants showed large spike, high thousand kernel weight (TKW), and mid-parent heterosis in plant height. The F_2_ plants with <70 cm in height, >45 g in TKW, and powdery mildew resistance were selected in 2005. One-half of seeds from each F_2_ individual differentiated as winter/spring growth habits at Guyuan experimental station (41°67′N and 115°69′E) of the Chinese Academy of Agricultural Sciences (CAAS) in Hebei in summer and 321 F_3_ plants with a winter growth habit were selected. In 2006, another half of the F_2_ seeds were evaluated for major agronomic traits at the Zhaoxian experimental station (37°87′N and 114°83′E) of CAAS in Hebei. Based on the overall performances at the two locations combined with molecular marker-assisted selection (MAS) at the *Vrn-D1* locus, 126 F_3_ lines carrying the recessive *vrn-D1vrn-D1* genotype were selected for evaluating their yield-related traits and the lodging resistance in the next generation. An F_5_ line RS03101-101-12-2 having a grain yield of 11.3% higher than the control Shi 4185 was selected in 2008. The F_6_ and F_7_ progenies of this line were further evaluated for yield-related traits, and finally, line RS03101-101-12-2-3-1 was advanced to yield assessment. It was commercially released as Lunxuan 103 in Hebei province in 2015 (Supplementary Figure 1), and subsequently in Shanxi, Shandong, and Tianjin in 2017, 2018, and 2019, respectively.

Shimai 12 (frost sensitivity) was crossed to Shijiazhuang 8 (frost tolerance) to generate the F_2_ and F_3_ populations. A natural population comprising 110 wheat cultivars was used to compare the difference in frost tolerance between the *Vrn-D1aVrn-D1a* genotype (50 spring cultivars) and the *vrn-D1vrn-D1* genotype (50 winter cultivars) (Supplementary Table 1).

### Developmental Morphology of Inflorescence and Response to Vernalization

Under the long-day condition (16 h light/8 h dark cycle), 20 germinated seeds of each genotype were sown in plastic pots, with two replicates. All genotypes were vernalized in a chamber at 4°C for 35 days and transferred to a light growth chamber under 16 h day length at 22°C and 8 h dark length at 18°C. Non-vernalized seedlings (0 days of cold treatment) as control were directly grown in the light growth chamber. For each treatment, three plants from each genotype were sampled for observing the developmental morphology of inflorescence using a stereoscopic microscope (Olympus, Tokyo, Japan). Days from sowing to the double ridge stage were used to measure the response to vernalization as described by Guo et al. (2015).

### Field Trials and Phenotypic Evaluation

#### Field Trials

During 2017/2018, 2018/2019, and 2020/2021 wheat growing seasons, field trials were conducted at the Shunyi Experimental Station (40°23′N and 116°57′E, 48 m above sea level) (SY) of CAAS in Beijing, the Zhaoxian Experimental Station (37°87′N and 114°83′E, 44 m above sea level) (ZX) of Shijiazhuang Academy of Agricultural and Forestry Sciences in Hebei province, the Dingxiang Experimental Station (38°50′N and 112°95′E, 866 m above sea level) (DX) of Shanxi Agricultural University (SXAU) in Shanxi, and the Taigu Experimental Station (37°42′N and 112°58′E, 799 m above sea level) (TG) of SXAU in Shanxi. The temperature information on each site is shown in Supplementary Figure 2. Field management, including fertilizers, irrigation, and management of weeds, diseases, and pests, was conducted as described previously (Meng et al., 2016).

Winter hardiness of the Shimai 12 × Shijiazhuang 8 F_2_ and F_3_ populations and a panel of wheat cultivars were evaluated at SY (2021SY), DX (2021DX), and TG (2021TG) during the 2020/2021 cropping season. All wheat entries were arranged in a randomized complete block design (RCBD) with two replicates. Each plot consisted of two rows, 2 m in length, 0.25 m apart, and 40 seeds in each row.

Lunxuan 103 and its parents were arranged in RCBD with six replicates, of which three replicates were used for evaluating winter frost tolerance and the rest three for investigating the agronomic traits. Winter frost tolerance was investigated at SY (2018SY) and ZX (2018ZX) during the 2017/2018 cropping season, and SY (2019SY) during the 2018/2019 cropping season, respectively. The agronomic traits were investigated at 2018SY, 2018ZX, and ZX (2019ZX) during the 2018/2019 cropping season. Each plot contained six rows, 6 m in length and 20 cm apart at site SY, and 7.5 m in length and 20 cm apart at each site, with the seeding rates 330, 300, and 300 per m^2^ at 2018/2019SY, 2018ZX, and 2019ZX environments, respectively.

### Winter Frost Tolerance

Winter frost tolerance was evaluated as described by the National Winter Wheat Regional Trials of China. At the three-leave stage, the total number of seedlings was investigated in 1 m double rows in a two-row plot or two sampling sites with 1-m double rows each selected along the diagonals in a six-row plot. When the daily mean temperature is <3°C, the total number of tillers was investigated in each environment. When new leaves were visible without the appearance of new tillers in spring, all seedlings at each sampling site were dug out for counting the number of winter-killed seedlings and tillers. Winter-killed seedling/tiller rates (%) were calculated by the number of winter-killed seedlings/tillers divided by the total number of seedlings/tillers before winter.

### Investigation of Agronomic Traits

The agronomic traits, including grain yield (kg/ha), kernel number per spike, spike number/m^2^, TKW (g), plant height (cm), and heading date (d), were assessed at 2018SY, 2018ZX, and 2019ZX environments, as previously described (Meng et al., 2016).

### Quantitative RT-PCR Analysis

Twenty germinated seeds of each genotype were sown in plastic pots and grown in a growth chamber for 14 d under the long-day condition (16 h light at 22°C/8 h dark at 18°C), with three replicates. To analyze the gene expression patterns, 14-d-old seedlings were grown at 4°C. Five leaves for each genotype were collected at 0 h, 3 h, 6 h, 12 h, 24 h, 2 d, 7 d, 14 d, 28 d, and 42 d. The total RNA extraction (TransGen Biotech Co., Ltd., Beijing, China) was conducted according to the manufacturer’s instructions. The first-strand of complementary DNA was synthesized using HiScript^®^ III All-in-one RT SuperMix Perfect for qPCR (Vazyme # R333, Vazyme Biotech Co., Ltd, Nanjing, China). The transcript levels of genes *Vrn-D1*, *TaCBF12*, *Wcs120*, and *Wcor410* were determined by quantitative RT-PCR (qRT-PCR) using a CFX Touch Real-Time PCR system (Bio-Rad, Hercules, CA, United States) with the gene-specific primers (Supplementary Table 2), and *Actin* was used as the internal reference gene. The reaction was carried out in a total volume of 20 μl consisting of 10 μl of 2 × *Taq* Pro Universal SYBR qPCR Master Mix (Vazyme Biotech Co., Ltd, Najing, China), 2 μl of cDNA (25 ng/μL), 0.5 μl of 10 μmol/L of each primer, and 7 μl of nuclease-free H_2_O. The amplification procedure was set as described by Xu K. et al. (2021). The relative expression level for each gene was calculated with the 2^–ΔΔCt^ method (Livak and Schmittgen, 2001). All data are presented as the means of three biological replicates.

### Marker and Genomic Composition Analyses

Twenty-one functional markers (Supplementary Table 3) specific for genes conferring plant height, vernalization, and photoperiod were used to differentiate alleles in Lunxuan 103 and its parents and association population (Supplementary Table 1). DNA amplification was carried out in a 20-μl reaction volume consisting of 1 μl of 50–100 ng/μl DNA, 1 μl of 10 μmol/L of each primer, 10 μl of 2 × *Taq* PCR Master Mix (Tsingke Biotechnology Co., Ltd., Beijing, China), and 7 μl of sterilized ddH_2_O. The thermal cycler reaction was set at 94°C for 5 min, followed by 35 cycles of denaturation at 94°C for 30 s, annealing at 50°C to 64°C for 30 s, extension at 72°C for 1 to 2 min, and a final extension step at 72°C for 10 min. PCR products were separated as described previously (Li T. et al., 2019).

Wheat cultivars were also genotyped using the Affymetrix Wheat 55K SNP array by China Golden Marker Biotechnology Co., Ltd., Beijing, China. After sample and marker quality control, data were processed using the Apt-genotype-axion, Probeset metrics, and Probeset classification modules by Axion Analysis Suite software (version 5.1.1) (Thermo Fisher Scientific-CN Co., Ltd., Shanghai, China).^1^ Sequences of SNPs were blasted against the Chinese Spring reference genome sequences (IWGSC RefSeq v1.0) to determine their chromosomal and physical locations.^2^ An R package was used to construct the genomic composition map (Beijing Biomarker Technologies Co., Ltd., Beijing, China). The GO (Gene Ontology) and KEGG (Kyoto Encyclopedia of Genes and Genomes) enrichment analyses were performed as described by Li et al. (2018).

### Statistical Analysis

Phenotypic differences in winter frost tolerance and agronomic traits among cultivars were tested using analysis of variance (ANOVA) in the SPSS software 26.0 (International Business Machines Corporation, Armonk, NY, United States), and multiple comparison analysis was performed using the least significant difference (LSD) test at *P* < 0.05. The differences in winter frost between genotypes in the association population were determined by the *t*-test in the SPSS software 26.0 (Wang et al., 2021).

## Results

### Inflorescence Development and Response to Vernalization

**FIGURE 1:**
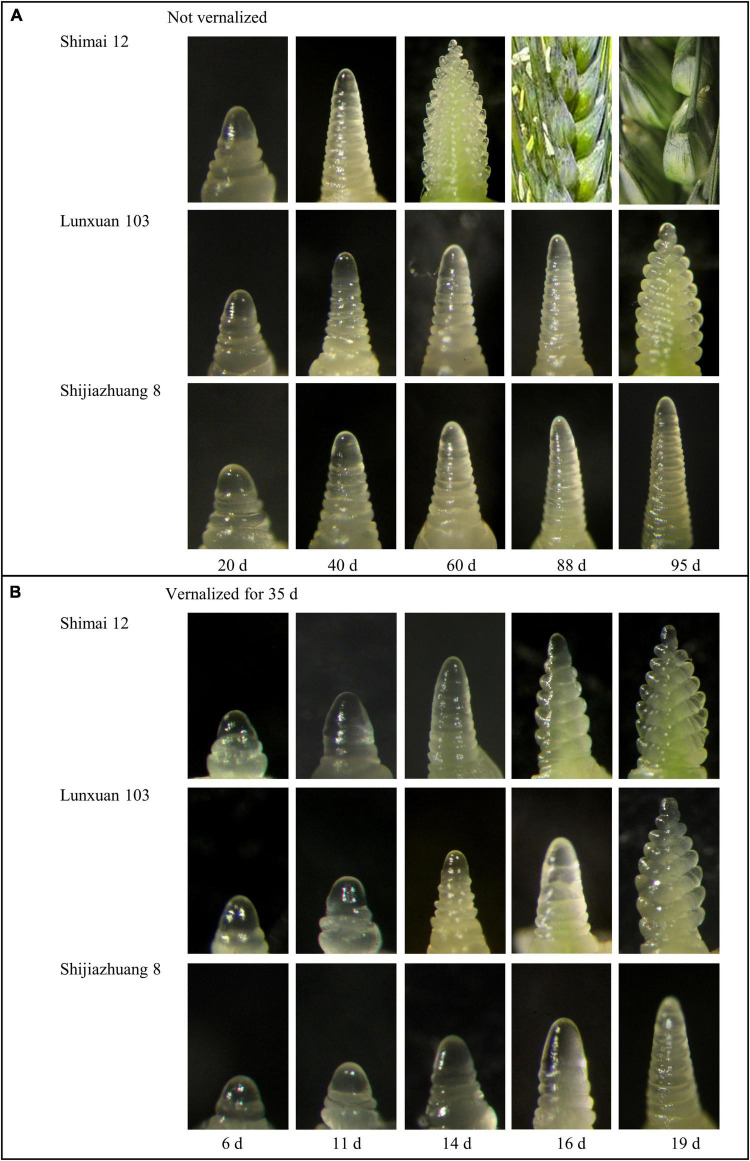
Inflorescence development of Lunxuan 103 and its parents, Shijiazhuang 8 and Shimai 12, with vernalization for 0 d **(A)** and 35 d **(B)** followed by growth under 16 h day length at 22°C and 8 h dark length at 18°C.

There was an obvious difference in spike differentiation among the three genotypes without vernalization (Figure 1A). Lunxuan 103 and its parents, Shimai 12 and Shijiazhuang 8, experienced the double ridge stage at 88, 40, and 95 d, respectively. The inflorescence of Shijiazhuang 8 elongated slowly and the spike development did not reach the double ridge stage until 95 d, at which Shimai 12 began grain-filling and Lunxuan 103 was at the glume differentiation stage (Figure 1A). When vernalized for 35 d, days to double ridge stage for Shimai 12, Lunxuan 103, and Shijiazhuang 8 were 14, 16, and 19 d, respectively (Figure 1B). The response to vernalization was 72 d for Lunxuan 103, 26 d for Shimai 12, and 76 d for Shijiazhuang 8. This shows that Lunxuan 103 is a typical winter cultivar with a strong response to vernalization (Supplementary Table 4).

### Winter Frost Tolerance of Lunxuan 103 in Comparison to the Parent Lines

**FIGURE 2:**
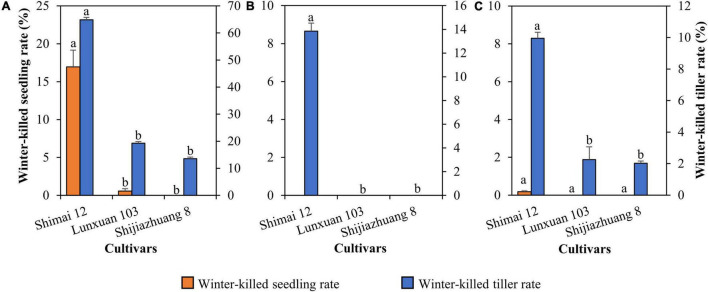
Comparison of freeze-killed seedlings and tillers for Lunxuan 103 and its parents Shijiazhuang 8 and Shimai 12 at 2018SY **(A)**, 2018ZX **(B)**, and 2019SY **(C)**. Multiple comparisons for each trait were performed using the least significant difference (LSD) test and different letters above the error bars indicate significant differences among cultivars at *P* < 0.05.

Compared to Shimai 12, the winter frost tolerance of Lunxuan 103 was significantly improved, and the rates of winter-killed seedling and winter-killed tiller were reduced by 16.4 and 45.6% (*P* < 0.05) at 2018SY, but no difference was observed between Lunxuan 103 and Shijiazhuang 8 (Figure 2A). There was a significant difference in winter-killed tiller rate between Lunxuan 103 and Shimai 12 at 2018ZX (Figure 2B), even though no dead seedlings were found for the three genotypes. The result in 2019SY was consistent with that in 2018ZX (Figure 2C).

### Relationship Between Different Alleles at the *Vrn-D1* Locus and Winter Frost Tolerance

**FIGURE 3:**
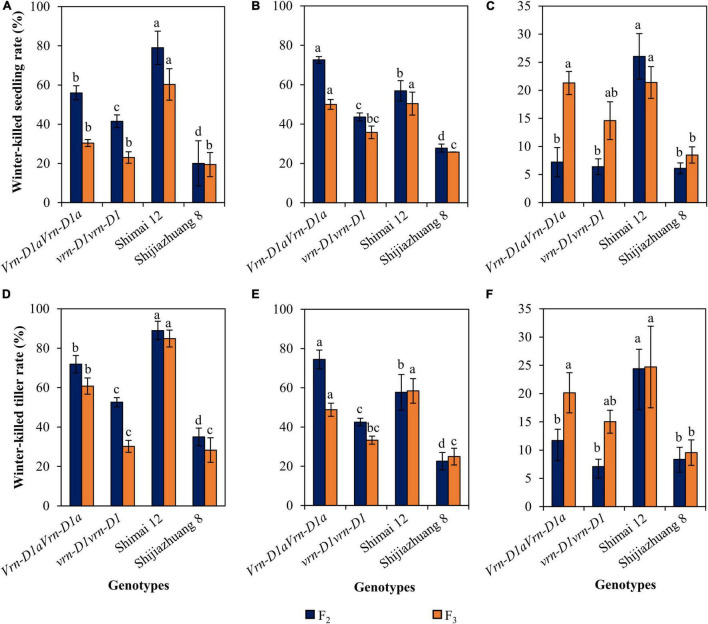
Comparison of winter-killed seedling and tiller rates between genotypes *vrn-D1vrn-D1* and *Vrn-D1aVrn-D1a* in the Shimai 12 × Shijiazhuang 8 F_2_ and F_3_ populations at 2021SY **(A,D)**, 2021DX **(B,E)**, and 2021TG **(C,F)**. In the F_2_ population, the numbers of genotypes *vrn-D1vrn-D1* and *Vrn-D1aVrn-D1a* are 764, 1091, and 1048, and 57, 108, and 102 at 2021SY, 2021DX, and 2021TG, respectively. In the F_3_ population, the numbers of genotypes, *vrn-D1vrn-D1* and *Vrn-D1aVrn-D1a*, are 22 and 8, respectively. Multiple comparisons were performed for each trait using the least significant difference (LSD) test and different letters above the error bars indicate significant differences among genotypes and parents at *P* < 0.05.

The progenies of cross Shimai 12 × Shijiazhuang 8 varied in winter frost tolerance at the three environments (Figure 3). Compared to the *Vrn-D1aVrn-D1a* genotype, the winter-killed seedling rate of the *vrn-D1vrn-D1* genotype was reduced by 14.5% (*P* < 0.05, 2021SY), 29.0% (*P* < 0.05, 2021DX), and 0.9% (2021TG) in the F_2_ generation, and 7.4% (2021SY), 14.2% (*P* < 0.05, 2021DX), and 6.7% (2021TG) in the F_3_ generation. The winter-killed tiller rate decreased by 19.3% (*P* < 0.05, 2021SY), 32.0% (*P* < 0.05, 2021DX), and 4.6% (2021TG) in the F_2_ generation, and 30.7% (*P* < 0.05, 2021SY), 15.5% (*P* < 0.05, 2021DX), and 5.1% (2021TG) in the F_3_ generation.

**FIGURE 4:**
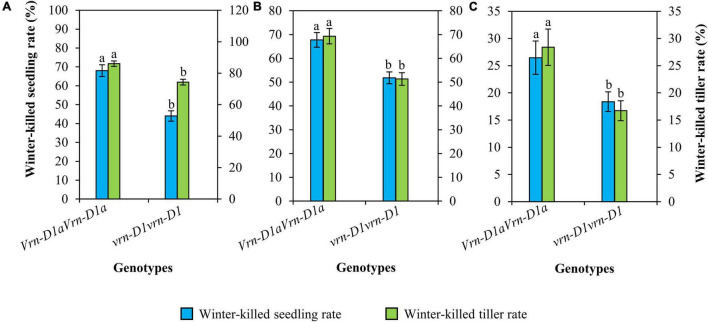
Comparison of winter frost tolerance between genotypes *vrn-D1vrn-D1* (*n* = 60) and *Vrn-D1aVrn-D1a* (*n* = 50) in the panel of wheat cultivars at 2021SY **(A)**, 2021DX **(B)**, and 2021TG **(C)**. Differences in winter frost between genotypes for each trait were determined by the *t*-test and different letters above the error bars indicate a significant difference at *P* < 0.05.

Cultivars carrying the *vrn-D1vrn-D1* genotype also showed significantly lower winter-killed seedling and winter-killed tiller rates than those carrying the *Vrn-D1aVrn-D1a* genotype (Figure 4). Compared to the *Vrn-D1aVrn-D1a* genotype, the winter-killed seedling rates of the *vrn-D1vrn-D1* genotype were significantly decreased by 24.0, 16.0, and 8.1%, and the winter-killed tiller rates were significantly reduced by 11.8, 18.0, and 11.7% at 2021SY (Figure 4A), 2021DX (Figure 4B), and 2021TG (Figure 4C), respectively.

### Effect of Winter Frost on Agronomic Traits

**FIGURE 5:**
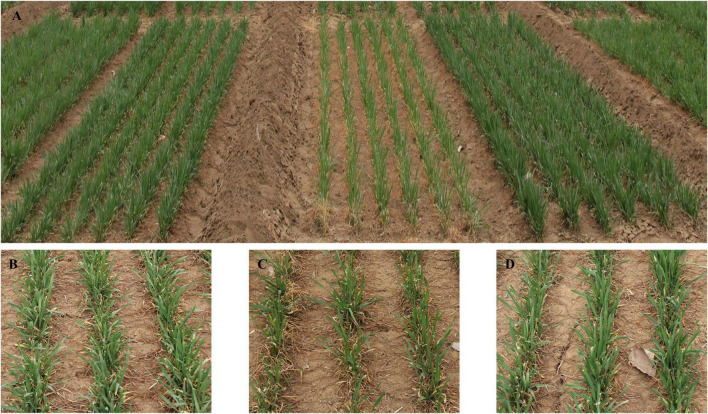
Performances of winter frost tolerance for wheat cultivar Lunxuan 103 and its parents Shijiazhuang 8 and Shimai 12 at 2018SY. **(A)** Plot performances of Shijiazhuang 8, Shimai 12, and Lunxuan 103 (from left to right) and partial performances of Shijiazhuang 8 **(B)**, Shimai 12 **(C)**, and Lunxuan 103 **(D)**.

**FIGURE 6:**
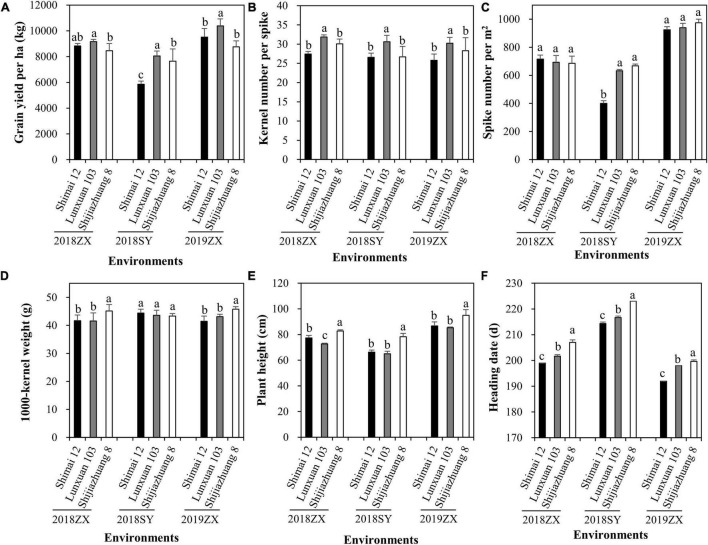
Comparison of grain yield per ha **(A)**, kernel number per spike **(B)**, spike number/m^2^ **(C)**, 1000-kernel weight **(D)**, plant height **(E)**, and heading date **(F)** among wheat cultivar Lunxuan 103 and its parents at the three environments. Multiple comparisons were performed using the least significant difference (LSD) test and different letters above the error bars indicate significant differences among cultivars in a single environment at *P* < 0.05.

The effect of winter frost on agronomic traits was evaluated in three environments. Severe winter frost occurred in 2018SY (Figure 5). Compared to Shijiazhuang 8 and Lunxuan 103, winter frost had large effects on Shimai 12 at 2018SY, which resulted in a significant decrease in grain yield (Figure 6A) and spike number/m^2^ (Figure 6C) and a slight increase in kernel number per spike (Figure 6B), but no effect on the other agronomic traits (Figures 6D–E). When no winter frost or light winter frost occurred, Shimai 12 exhibited a higher grain yield than Shijiazhuang 8 at 2019ZX and 2018ZX (Figure 6A). Lunxuan 103 showed higher grain yield, more kernel number per spike (*P* < 0.05), shorter plant height than its parents, and an earlier heading date than Shijiazhuang 8 in all environments (Figure 6).

### Expression of Vernalization Gene *Vrn-D1* and Cold-Responsive Genes

**FIGURE 7:**
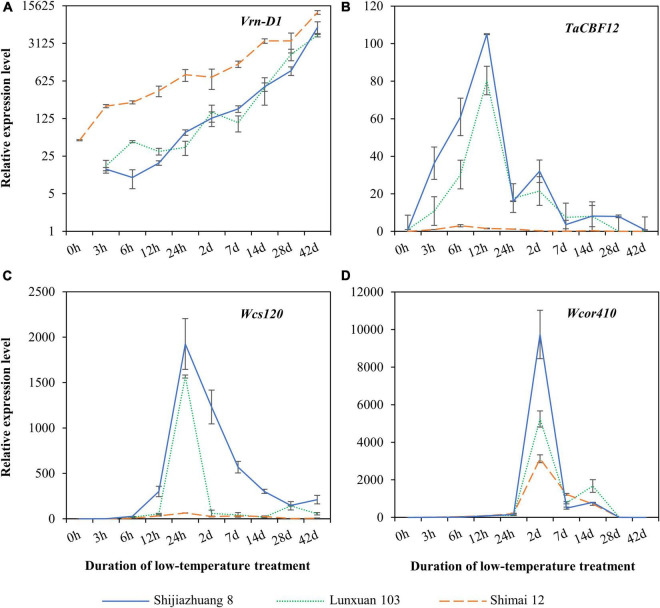
Expression analysis of vernalization gene *Vrn-D1* **(A)** and cold-responsive genes *TaCBF12* **(B)**, *Wcs120* **(C)**, and *Wcor410* **(D)** in Lunxuan 103 and its parents Shijiazhuang 8 and Shimai 12.

Expression patterns of the vernalization gene *Vrn-D1* and the three cold-responsive genes were quantitatively compared among Lunxuan 103 and its parents, Shimai 12 and Shijiazhuang 8 (Figure 7). A dramatically different expression pattern was observed in *Vrn-D1* among the three cultivars, and Shimai 12 showed significantly higher expression levels than Lunxuan 103 and Shijiazhuang 8 in all treatments (Figure 7A). Under the non-vernalization condition (0 h), the transcriptional accumulation of *Vrn-D1* was observed in Shimai 12, followed by a steady increase until 42 d. Conversely, Shijiazhuang 8 and Lunxuan 103 had no *Vrn-D1* expression at 0 h followed by a slow increase from 3 h to 14 d, and a sudden increase from 28 to 42 d (Figure 7A). The transcripts of the transcriptional factor gene *TaCBF12* were detected at a low level in the three cultivars under the non-vernalized condition. The expression of *TaCBF12* increased within 3 h after exposure of the seedlings at 4°C, peaked at 12 h, and declined thereafter (Figure 7B). A high transcript level of *TaCBF12* was detected in the frost-tolerant cultivars Shijiazhuang 8 and Lunxuan 103 at 12 h, but not in the frost-sensitive cultivar Shimai 12. A similar expression pattern was observed for *Wcs120*, and Shijiazhuang 8 and Lunxuan 103 showed significantly higher expression levels at 24 h than Shimai 12 (Figure 7C). The cold-responsive/late-embryogenesis-abundant (*Cor/Lea*) gene *Wcor410* was weakly expressed under the non-vernalized condition and dramatically accumulated at 2 d of low-temperature treatment in the three cultivars. Shijiazhuang 8 showed the highest transcript level of *Wcor410* at 2 d, followed by Lunxuan 103, and Shimai 12 had a relatively low expression level (Figure 7D).

### Molecular Marker and Genomic Composition Analyses of Lunxuan 103 and Its Parents

**FIGURE 8:**
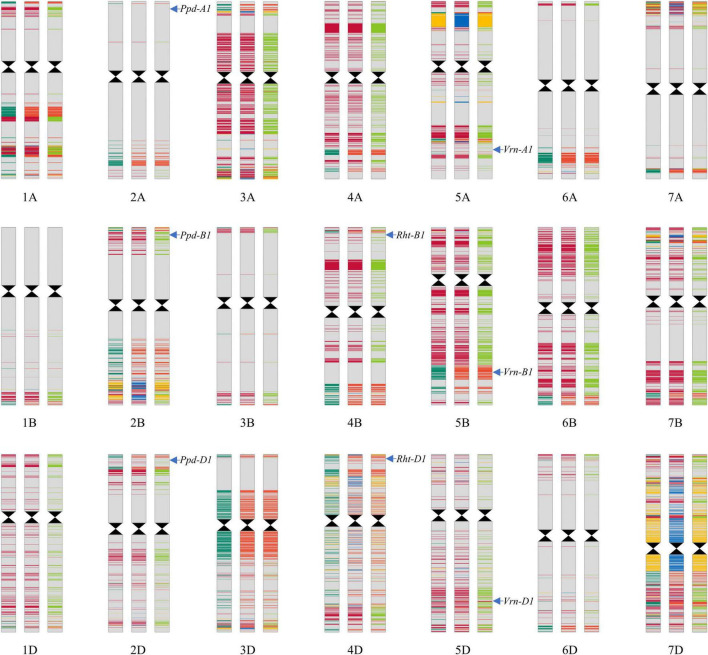
Genotypic patterns of Lunxuan 103 (central panel) and its parents, Shimai 12 (the left panel) and Shijiazhuang 8 (right panel), are based on the Wheat 55K SNP array. The gray color represents the identical SNPs shared by the three genotypes. Dark red, light red, and yellow colors represent the common SNPs shared by Lunxuan 103 and Shimai 12, Lunxuan 103 and Shijiazhuang 8, and Shimai 12 and Shijiazhuang 8, respectively. Dark green, blue, and light green colors represent the unique SNPs of Shimai 12, Lunxuan 103, and Shijiazhuang 8, respectively.

Lunxuan 103 and its parents shared the same alleles at the loci associated with plant height, vernalization, and photoperiod, except for the vernalization gene *Vrn-D1* (Figure 8). At the *Vrn-D1* locus, Lunxuan 103 carried the same recessive *vrn-D1* allele as Shijiazhuang 8, whereas Shimai 12 contained the dominant *Vrn-D1a* allele.

Based on the Wheat 55K SNP array, a total of 43,439 high-quality SNPs were used to construct a genomic composition map using the information on the physical locations of SNPs (Figure 8). Among them, 74.4% (32,331) of SNPs were identical among the three genotypes, and 90.3% (39,209) and 81.4% (35,367) SNPs of Lunxuan 103 were common with Shimai 12 and Shijiazhuang 8, respectively. The relative genetic contributions of the two parents, Shimai 12 and Shijiazhuang 8, to Lunxuan 103 were 52.6 and 47.4%, respectively (Supplementary Table 5). In addition, 2.7% (1194) SNPs were unique to Lunxuan 103. The common SNPs shared by the three genotypes were mainly associated with binding, catalytic activity, transporter activity, structural molecule activity, and transcription factor activity (Supplementary Figure 3 and Supplementary Table 6). The unique SNPs to Lunxuan 103 were mainly associated with the developmental process and multicellular organismal process (Supplementary Figure 3), and these genes were mainly involved in 33 metabolic pathways, in which four pathways were significant for the unique genes of Lunxuan 103 (Supplementary Tables 6, 7).

## Discussion

Many studies have demonstrated that the recessive *vrn-1* allele is associated with frost tolerance (Sutka, 1981; Veisz and Sutka, 1989; Tóth et al., 2003; Koemel et al., 2004; Babben et al., 2018). Shimai 12 and Shijiazhuang 8 share identical alleles at known vernalization and photoperiod loci, except *Vrn-D1*, but differ in winter hardiness. Shijiazhuang 8 with the recessive *vrn-D1* allele has a winter growth habit, whereas Shimai 12 with the dominant *Vrn-D1a* allele has the typical characteristic of spring cultivars (Zhang et al., 2010, 2012). We aimed to select the progenies with the homozygous *vrn-D1vrn-D1* genotype at the *Vrn-D1* locus during the development of Lunxuan 103. As a result, the winter frost tolerance of Lunxuan 103 was significantly improved. Based on the analysis of the relationship between the alleles at the *Vrn-D1* locus and the frost tolerance in the genetic population and different wheat cultivars, it was confirmed that the recessive *vrn-D1* allele can improve wheat winter frost tolerance compared to the dominant *Vrn-D1a* allele.

Based on the observation of spike differentiation and response to vernalization, Lunxuan 103 and Shijiazhuang 8 required 48 to 55 d more than Shimai 12 to reach the double ridge stage, and the difference was 2–5 d under vernalization for 35 d, suggesting that Lunxuan 103 and Shijiazhuang 8 have winter growth habit with strong vernalization requirement. Hence, Lunxuan 103 and Shijiazhuang 8 need a long period at a nonfreezing low temperature to meet the vernalization requirement (Dhillon et al., 2010). Vernalization is a cumulative process (Xu and Chong, 2018), which delays the wheat’s shoot apical meristem development and accumulates low-temperature tolerance until vernalization saturation (Fowler et al., 1996). This might explain that a high level of frost tolerance is observed in Lunxuan 103 and Shijiazhuang 8, but not in Shimai 12.

The frost tolerance wheat cultivars showed higher transcript levels in *CBF* and the *COR*/*LEA* genes than those in frost-sensitive cultivars (Motomura et al., 2013; Todorovska et al., 2014; Shi et al., 2018). *Wcs120* and *Wcor410* play significant roles in frost tolerance and had higher expression levels in the winter tolerant cultivars than in the sensitive spring wheat cultivars (Ganeshan et al., 2008; Motomura et al., 2013; Vítámvás et al., 2019). In the clustered *CBF* copies, *TmCBF12* is a candidate for wheat *Fr-Am2* (Knox et al., 2008). The transcript levels of *Wcs120*, *Wcor410*, and *TaCBF12* were significantly higher in winter wheat cultivars Lunxuan 103 and Shijiazhuang 8 than in spring wheat cultivar Shimai 12. This finding is in agreement with the results of the previous studies (Ganeshan et al., 2008; Motomura et al., 2013). These observations suggest that the better winter frost tolerance of Lunxuan 103 is the result of an ability to maintain low-temperature-tolerance genes in an upregulated state.

The expression of *vrn-1* or its induced genes can downregulate the *CBF/COR* genes (Linmin and Fowler, 2006; Shi et al., 2018), which inversely affected the level of frost tolerance (Kobayashi et al., 2005; Stockinger et al., 2007; Shi et al., 2018). Wheat cultivars with the dominant *Vrn* alleles had winter freezing tolerance than those carrying the corresponding recessive alleles (Koemel et al., 2004; Linmin and Fowler, 2006; Reddy et al., 2006). The previous study showed that a higher level of expression of *Vrn-D1* was observed in the spring accessions compared to the facultative or winter ones under the non-vernalized condition (Zhang et al., 2012). It can be inferred that Shimai 12 is more sensitive to low temperature than Shijianzhuang 8, and Lunxuan 103 is attributed to its higher expression of *Vrn-D1* at the vegetative stage. The improvement of freezing tolerance in Lunxuan 103 suggests that the allelic variation in the *Vrn-D1* locus is sufficient to explain the differences in its freezing tolerance.

Grain yield is a high priority in most wheat breeding programs. Lunxuan 103 showed a higher grain yield than its parents regardless of the occurrence of winter frost. Based on the high-quality SNPs, the two parents contributed similar proportions of genetic loci to Lunxuan 103, suggesting that a high frequency of genetic recombination occurred in Lunxuan 103. However, Shimai 12 showed higher relative genetic contributions to Lunxuan 103 than Shijiazhuang 8 on chromosomes 3A, 4A, 5B, 6B, 1D, 2D, and 5D, whereas Shijiazhuang 8 had a higher proportion of contributions on chromosomes 3D and 6A. Previous studies have identified many QTLs associated with grain yield-related traits on the above-mentioned chromosomes. For example, the main-effect QTL *QGns.cau-4A.4* controlling kernel number per spike was repeatedly identified on chromosome 4A and explained a range of phenotypic variation from 5.0 to 21.4% (Gao et al., 2015; Cui et al., 2017; Guan et al., 2018). A major QTL *QYld.tam-2D* for grain yield was located on chromosome 2D, with the phenotypic variation explained by 12.9–21.9% (Mason et al., 2013; Yu et al., 2018). It can be inferred that the better yield performance of Lunxuan 103 than its parents might result from gene pyramiding or interaction between the favorable loci from the two parents.

In this study, the 1194 specific SNPs to Lunxuan 103 could not be associated directly with winter frost due to the limitation of the gene annotations. However, these specific SNPs would be developed into specific markers for Lunxuan 103 for accurate identification during the commercialization of wheat breeding (Li et al., 2018).

In conclusion, the winter frost tolerance of spring wheat Shimai 12 has been significantly improved by introducing the *vrn-D1* allele at the *Vrn-D1* locus from winter cultivar Shijiazhuang 8, as demonstrated in the derived cultivar Lunxuan 103. Furthermore, Lunxuan 103 shows a higher grain yield potential than its parents. This study provides a successful example of improvement in winter frost tolerance by regulating vernalization responses in common wheat.

## Data Availability Statement

The datasets presented in this study can be found in online repositories. The names of the repository/repositories and accession number(s) can be found in the article/Supplementary Material.

## Author Contributions

YZ and HL designed the experiments. HZ, XX, JG, YH, XD, TL, JH, YQ, LQY, CM, HWL, and LY performed the experiments. HZ, XX, YZ, and HL analyzed the data and wrote the manuscript. All authors reviewed the manuscript.

## Conflict of Interest

The authors declare that the research was conducted in the absence of any commercial or financial relationships that could be construed as a potential conflict of interest.

## Publisher’s Note

All claims expressed in this article are solely those of the authors and do not necessarily represent those of their affiliated organizations, or those of the publisher, the editors and the reviewers. Any product that may be evaluated in this article, or claim that may be made by its manufacturer, is not guaranteed or endorsed by the publisher.

## Footnotes

1
https://www.thermofisher.cn/cn/zh/home/life-science/microarray-analysis/microarray-analysis-instruments-software-services/microarray-analysis-software.html

2
http://www.wheatgenome.org/

## Supplementary Material

Supplementary Figure 1 Breeding procedure of wheat cultivar Lunxuan 103.

Supplementary Figure 2 Average daily maximum **(A)** and minimum **(B)** temperatures at five environments during wheat cropping seasons (from October of the first year to June of the next year).

Supplementary Figure 3 Category analysis of the genes identified by the common SNPs (CG), specific SNPs of Shimai 12 to Lunxuan 103 (SG-SM12), specific SNPs of Shijiazhuang 8 to Lunxuan 103 (SG-SJZ8), and specific SNPs to Lunxuan 103 (SG-LX103).
