# Supplementary figures and images for: Association of the Recessive Allele vrn-D1 With Winter Frost Tolerance in Bread Wheat
Source: Front Plant Sci. 2022 Jun 6;13:879768. doi: 10.3389/fpls.2022.879768 (PMC9207342; doi:10.3389/fpls.2022.879768)

## Supplementary Figure 1

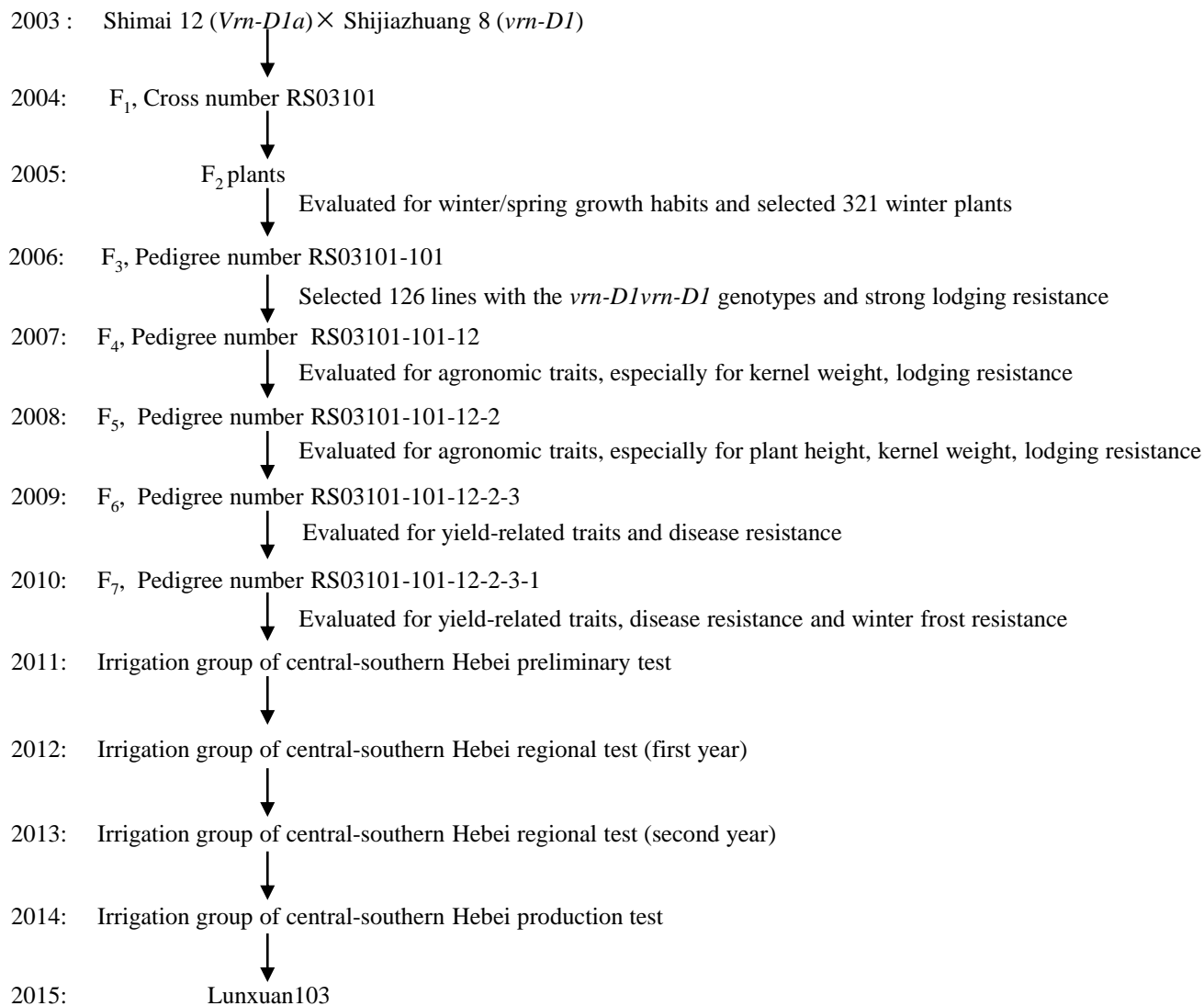

Supplementary Figure 2

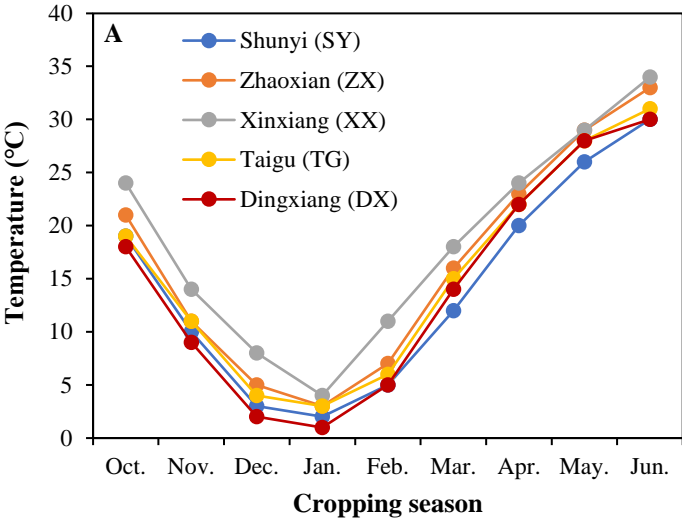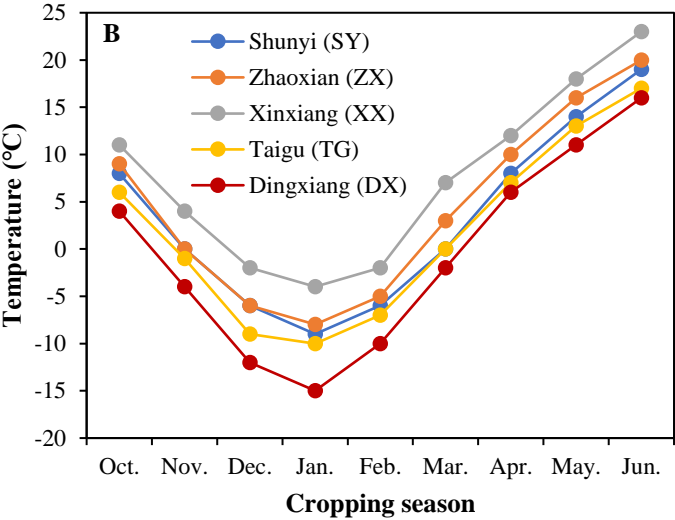

Supplementary Figure 3

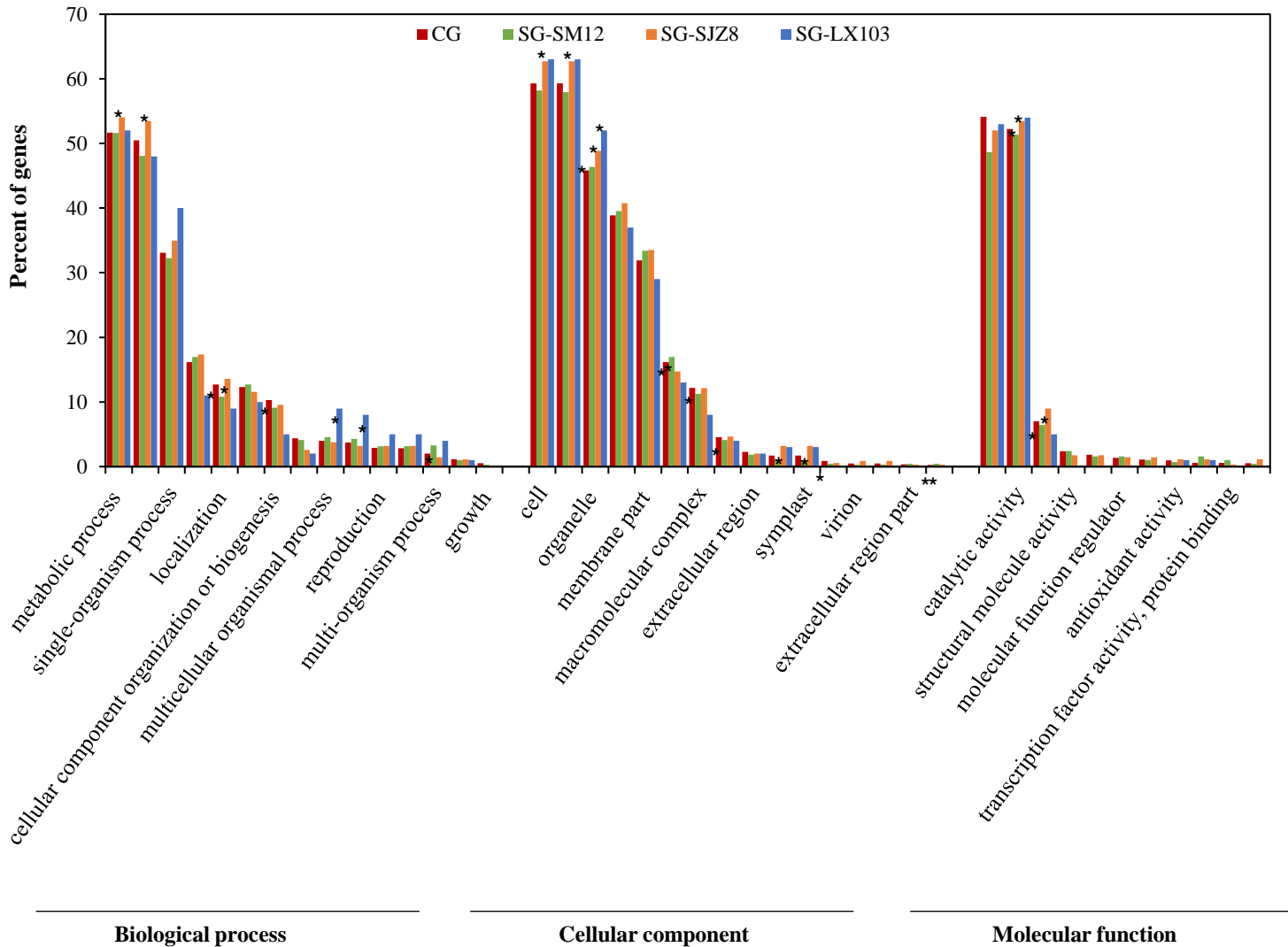

Supplement: Supplementary Figure 1 — Breeding procedure of wheat cultivar Lunxuan 103. [file Data_Sheet_1.PDF]
